# Bis{tris­[3-(2-pyrid­yl)-1*H*-pyrazole]manganese(II)} dodeca­molybdo(V,VI)phosphate hexa­hydrate

**DOI:** 10.1107/S160053681000320X

**Published:** 2010-01-30

**Authors:** Lujiang Hao, Chunling Ma, Jianghui Chen, Xiaofei Zhang, Xiutang Zhang

**Affiliations:** a College of Food and Biological Engineering, Shandong Institute of Light Industry, Jinan 250353, People’s Republic of China; b Advanced Material Institute of Research, Department of Chemistry and Chemical Engineering, ShanDong Institute of Education, Jinan 250013, People’s Republic of China; c College of Chemistry and Chemical Engineering, Liaocheng University, Liaocheng 252059, People’s Republic of China

## Abstract

The asymmetric unit of the title compound, [Mn(C_8_H_7_N_3_)_3_]_2_[PMo_12_O_40_]·6H_2_O, consists of a complex [Mn(C_8_H_7_N_3_)_3_]^2+^ cation, half of a mixed-valent Mo^V,VI^ α-Keggin-type [PMo_12_O_40_]^4−^ heteropolyanion, and three uncoordinated water mol­ecules. The Mn^2+^ cation is surrounded by six N atoms from three chelating 3-(2-pyrid­yl)-1*H*-pyrazole ligands in a distorted octa­hedral coordination. In the heteropolyanion, two O atoms of the central PO_4_ group (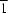 symmetry) are equally disordered about an inversion centre. N—H⋯O and O—H⋯O hydrogen bonding between the cations, anions and the uncoordinated water mol­ecules leads to a consolidation of the structure.

## Related literature

For general background to polyoxometalates, see: Pope & Müller (1991 ▶). For polyoxometalates modified with amines, see: Zhang, Dou *et al.* (2009 ▶); Zhang, Wei *et al.* (2009 ▶). For the structure and chemistry of reduced heteropolyanions with composition [PMo_12_O_40_]^4−^, see: Artero & Proust (2000 ▶); Kurmoo *et al.* (1998 ▶).; Niu *et al.* (1999 ▶). For the role of amines in hydro­thermal synthesis, see: Yang *et al.* (2003 ▶).
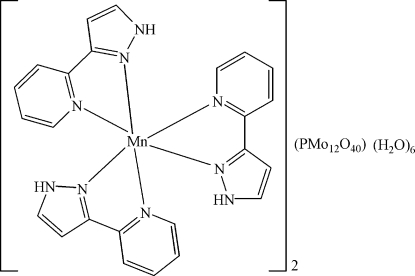


## Experimental

### 

#### Crystal data

[Mn(C_8_H_7_N_3_)_3_]_2_[PMo_12_O_40_]·6H_2_O
*M* *_r_* = 2911.22
Monoclinic, 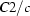

*a* = 18.897 (4) Å
*b* = 16.360 (3) Å
*c* = 27.615 (6) Å
β = 104.90 (3)°
*V* = 8250 (3) Å^3^
*Z* = 4
Mo *K*α radiation
μ = 2.18 mm^−1^
*T* = 293 K
0.12 × 0.10 × 0.08 mm

#### Data collection

Bruker APEXII CCD diffractometer
Absorption correction: multi-scan (*SADABS*; Bruker, 2001 ▶) *T*_min_ = 0.780, *T*_max_ = 0.845
15879 measured reflections
7042 independent reflections
5890 reflections with *I* > 2σ(*I*)
*R*_int_ = 0.019

#### Refinement

*R*[*F* ^2^ > 2σ(*F* ^2^)] = 0.035
*wR*(*F* ^2^) = 0.103
*S* = 1.00
7042 reflections
610 parameters
H-atom parameters constrained
Δρ_max_ = 1.52 e Å^−3^
Δρ_min_ = −0.59 e Å^−3^

### 

Data collection: *APEX2* (Bruker, 2004 ▶); cell refinement: *SAINT-Plus* (Bruker, 2001 ▶); data reduction: *SAINT-Plus*; program(s) used to solve structure: *SHELXS97* (Sheldrick, 2008 ▶); program(s) used to refine structure: *SHELXL97* (Sheldrick, 2008 ▶); molecular graphics: *SHELXTL* (Sheldrick, 2008 ▶); software used to prepare material for publication: *SHELXTL*.

## Supplementary Material

Crystal structure: contains datablocks global, I. DOI: 10.1107/S160053681000320X/wm2301sup1.cif

Structure factors: contains datablocks I. DOI: 10.1107/S160053681000320X/wm2301Isup2.hkl

Additional supplementary materials:  crystallographic information; 3D view; checkCIF report

## Figures and Tables

**Table 1:** Selected bond lengths (Å)

Mn1—N8	2.224 (6)
Mn1—N5	2.224 (5)
Mn1—N2	2.250 (5)
Mn1—N4	2.259 (6)
Mn1—N1	2.260 (5)
Mn1—N7	2.283 (5)
P1—O21*A*^i^	1.495 (7)
P1—O21*B*^i^	1.519 (7)
P1—O19*B*^i^	1.531 (6)
P1—O19*A*^i^	1.562 (6)

Symmetry code: (i) 
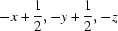
.

**Table 2:** Hydrogen-bond geometry (Å, °)

*D*—H⋯*A*	*D*—H	H⋯*A*	*D*⋯*A*	*D*—H⋯*A*
N9—H9*A*⋯O1*W*	0.86	1.96	2.786 (9)	160
N6—H6⋯O2*W*	0.86	2.10	2.951 (13)	171
N3—H3*A*⋯O17^ii^	0.86	1.97	2.814 (7)	165

Symmetry code: (ii) 
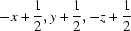
.

**Fig. 1.**
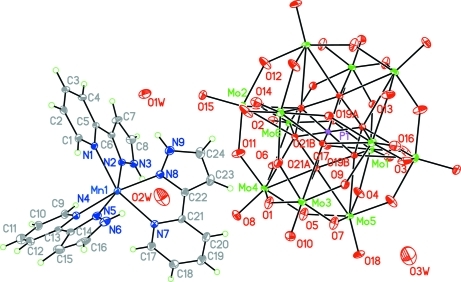
The building blocks of the title compound with the atom-labelling scheme. Displacement ellipsoids are drawn at the 30% probability level; H atoms are given as spheres of arbitrary radius.

**Fig. 2.**
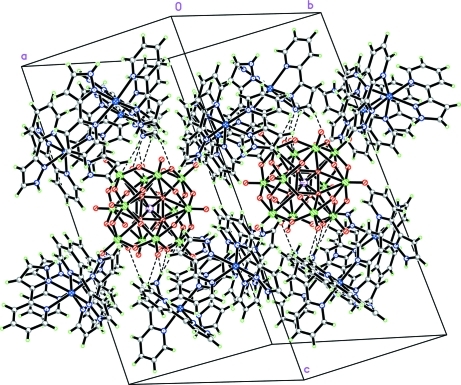
The crystal packing of the title compound, displayed with N—H···O and O—H···O hydrogen bonds as dashed lines. Water molecules have been omitted for clarity.

## supplementary crystallographic information

### Comment

The design and synthesis of polyoxometalates has attracted continuous research interest not only because of their appealing structural and topological novelties, but also due to their interesting optical, electronic, magnetic, and catalytic properties, as well as their potential medical applications (Pope & Müller, 1991). In our research group, organic amines, such as 3-(2-pyridyl)pyrazole and pyrazine, are used to effectively modify polyoxomolybdates under hydrothermal condictions (Zhang, Dou *et al.*, 2009; Zhang, Wei *et al.*, 2009). Here, we describe the synthesis and structural characterization of the title compound.

As shown in Figure 1, the asymmetric unit of the title compound consists of three subunits, *viz.* of a complex [Mn(C_8_H_7_N_3_)_3_]^2+^ cation, a heteropolyanion [PMo_12_O_40_]^4-^ and of three uncoordinated water molecules. The Mn(II) ion is distorted octahedrally coordinated by six N atoms from three chelating 3-(2-pyridyl)-1*H*-pyrazole ligands. The Mn—N bond lengths are in the range of 2.224 (6)—2.283 (5) Å.

The heteropolyanion [PMo_12_O_40_]^4-^ is an one electron-reduced derivative of [PMo_12_O_40_]^3-^, similar to other reported representatives (Artero & Proust, 2000; Kurmoo *et al.*, 1998; Niu *et al.*, 1999). The employed organic ligand appears to adjust the pH value, and additionally supplies reducing electrons, which is a commonly observed feature of hydrothermal syntheses when organic amines are used to prepare various hybrid materials, zeolites or metal phosphates (Yang *et al.*, 2003).

In the Keggin-type heteropolyanion, each Mo atom is surrounded by six O atoms and the P atom is located at the centre of the anion. There are four kinds of O atoms present in the anion according to their coordination environments: O*_a_* (O atoms in the disordered PO_4_ tetrahedron), O*_b_* (bridging O atoms between two triplet groups of MoO_6_ octahedra), O*_c_* (bridging O atoms within one triplet group of MoO_6_ octahedra) and O*_d_* (terminal O atoms). The P—O bond distances are in the normal range of 1.495 (7)—1.562 (6) Å. The Mo—O bond distances vary widely from 1.644 (4) to 2.517 (6) Å. The shortest Mo—O bonds are in the range of 1.644 (4)—1.666 (4) Å for the terminal oxygen atoms. The longest Mo—O lengths are in the range of 2.455 (7)—2.517 (6) Å for those oxygen atoms connected with both Mo and P atoms.

N—H···O and O—H···O hydrogen bonding between the cationic and anionic moieties and the uncoordinated water molecules leads to a consolidation of the structure (Fig. 2; Table 2).

### Experimental

A mixture of 3-(2-pyridyl)-1*H*-pyrazole (0.5 mmoL 0.07 g), sodium molybdate (0.4 mmoL, 0.10 g), manganese(II) sulfate monohydrate (0.3 mmol, 0.05 g), and dipotassium hydrogenphosphate (0.22 mmol, 0.05 g) in 10 ml distilled water was sealed in a 25 ml Teflon-lined stainless steel autoclave and was kept at 433 K for three days. Pink crystals suitable for the X-ray experiment were obtained. Anal. C_48_H_54_Mn_2_Mo_12_N_18_O_46_P: C, 19.80; H, 1.82; N, 8.66. Found: C, 19.71; H, 1.74; N, 8.58 %. IR(cm-1): 3456, 1676, 1605, 1433, 1363, 1306, 1015, 959, 773, 739, 694, 942.

TGA curve shows that the lattice water molecules and the organic ligands seperate above 372 and 683 K, respectively. The overall thermal decomposition process can be described by the followed equation: 4C_48_H_54_Mn_2_Mo_12_N_18_O_46_P + 325O_2_ = 108H_2_O + 192CO_2_ + 36N_2_O_5_ + 8MnO + 2P_2_O_5_ + 48MoO_3_

### Refinement

All hydrogen atoms bound to aromatic carbon atoms were refined in calculated positions using a riding model with a C—H distance of 0.93 Å and *U*_iso_ = 1.2*U*_eq_(C). Hydrogen atoms attached to aromatic N atoms were refined with a N—H distance of 0.86 Å and *U*_iso_ = 1.2*U*_eq_(N). The hydrogen atoms of the three uncoordinated water molecules could not be located unambiguously from difference Fourier maps, probably due to disorder of the water molecules. Thus the structure was refined without the H atoms of the water molecules (which includes the water O atoms O1W, O2W, O3W). In the PO_4_ unit, the two oxygen atoms (O19 and O21) are equally disordered about the inversion centre. In the final difference Fourier map the highest peak is 2.86 Å from atom O2w and the deepest hole is 1.09 Å from atom O9. The highest peak is located in the voids of the crystal structure and may be associated with an additional water molecule. However, refinement of this position did not result in a reasonable model. Hence this position was also excluded from the final refinement.

### Crystal data

**Table:**

[Mn(C_8_H_7_N_3_)_3_]_2_[PMo_12_O_40_]·6H_2_O	*F*(000) = 5620
*M**_r_* = 2911.22	*D*_x_ = 2.344 Mg m^−^^3^
Monoclinic, *C*2/*c*	Mo *K*α radiation, λ = 0.71073 Å
Hall symbol: -C 2yc	Cell parameters from 7042 reflections
*a* = 18.897 (4) Å	θ = 2.9–25.0°
*b* = 16.360 (3) Å	µ = 2.18 mm^−^^1^
*c* = 27.615 (6) Å	*T* = 293 K
β = 104.90 (3)°	Block, pink
*V* = 8250 (3) Å^3^	0.12 × 0.10 × 0.08 mm
*Z* = 4	

### Data collection

**Table:**

Bruker APEXII CCD diffractometer	7042 independent reflections
Radiation source: fine-focus sealed tube	5890 reflections with *I* > 2σ(*I*)
graphite	*R*_int_ = 0.019
phi and ω scans	θ_max_ = 25.0°, θ_min_ = 2.9°
Absorption correction: multi-scan (*SADABS*; Bruker, 2001)	*h* = −19→22
*T*_min_ = 0.780, *T*_max_ = 0.845	*k* = −19→18
15879 measured reflections	*l* = −32→15

### Refinement

**Table:**

Refinement on *F*^2^	Primary atom site location: structure-invariant direct methods
Least-squares matrix: full	Secondary atom site location: difference Fourier map
*R*[*F*^2^ > 2σ(*F*^2^)] = 0.035	Hydrogen site location: inferred from neighbouring sites
*wR*(*F*^2^) = 0.103	H-atom parameters constrained
*S* = 1.00	*w* = 1/[σ^2^(*F*_o_^2^) + (0.058*P*)^2^ + 43.0549*P*] where *P* = (*F*_o_^2^ + 2*F*_c_^2^)/3
7042 reflections	(Δ/σ)_max_ = 0.001
610 parameters	Δρ_max_ = 1.52 e Å^−^^3^
0 restraints	Δρ_min_ = −0.59 e Å^−^^3^

### Special details

**Table:**

Geometry. All esds (except the esd in the dihedral angle between two l.s. planes) are estimated using the full covariance matrix. The cell esds are taken into account individually in the estimation of esds in distances, angles and torsion angles; correlations between esds in cell parameters are only used when they are defined by crystal symmetry. An approximate (isotropic) treatment of cell esds is used for estimating esds involving l.s. planes.
Refinement. Refinement of *F*^2^ against ALL reflections. The weighted *R*-factor wR and goodness of fit *S* are based on *F*^2^, conventional *R*-factors *R* are based on *F*, with *F* set to zero for negative *F*^2^. The threshold expression of *F*^2^ > σ(*F*^2^) is used only for calculating *R*-factors(gt) etc. and is not relevant to the choice of reflections for refinement. *R*-factors based on *F*^2^ are statistically about twice as large as those based on *F*, and *R*- factors based on ALL data will be even larger.

### Fractional atomic coordinates and isotropic or equivalent isotropic displacement parameters (Å^2^)

**Table:**

	*x*	*y*	*z*	*U*_iso_*/*U*_eq_	Occ. (<1)
C1	0.2008 (4)	0.9012 (4)	0.0945 (2)	0.0468 (15)	
H1	0.2435	0.8957	0.0840	0.056*	
C2	0.1413 (4)	0.9354 (4)	0.0625 (2)	0.0514 (16)	
H2	0.1441	0.9547	0.0314	0.062*	
C3	0.0778 (4)	0.9412 (4)	0.0766 (2)	0.0520 (16)	
H3	0.0364	0.9640	0.0551	0.062*	
C4	0.0755 (3)	0.9131 (4)	0.1228 (2)	0.0484 (15)	
H4	0.0322	0.9158	0.1328	0.058*	
C5	0.1377 (3)	0.8807 (3)	0.1543 (2)	0.0365 (12)	
C6	0.1409 (3)	0.8535 (4)	0.2053 (2)	0.0404 (13)	
C7	0.0856 (4)	0.8496 (5)	0.2303 (2)	0.064 (2)	
H7	0.0365	0.8635	0.2181	0.076*	
C8	0.1203 (4)	0.8206 (5)	0.2770 (2)	0.067 (2)	
H8	0.0985	0.8115	0.3032	0.080*	
C9	0.3304 (4)	0.9890 (4)	0.2657 (3)	0.0570 (17)	
H9	0.2996	0.9679	0.2840	0.068*	
C10	0.3564 (5)	1.0653 (5)	0.2751 (3)	0.069 (2)	
H10	0.3428	1.0958	0.2997	0.083*	
C11	0.3992 (6)	1.0970 (7)	0.2513 (4)	0.098 (3)	
H11	0.4173	1.1495	0.2591	0.118*	
C12	0.4172 (5)	1.0565 (6)	0.2166 (4)	0.082 (3)	
H12	0.4473	1.0801	0.1986	0.098*	
C13	0.3917 (3)	0.9775 (4)	0.2058 (3)	0.0552 (17)	
C14	0.4124 (3)	0.9290 (5)	0.1680 (3)	0.0552 (17)	
C15	0.4646 (5)	0.9425 (7)	0.1395 (4)	0.091 (3)	
H15	0.4956	0.9871	0.1408	0.109*	
C16	0.4578 (4)	0.8683 (7)	0.1073 (3)	0.088 (3)	
H16	0.4837	0.8562	0.0838	0.105*	
C17	0.4104 (4)	0.7489 (5)	0.2977 (3)	0.0660 (19)	
H17	0.4144	0.8031	0.3083	0.079*	
C18	0.4495 (5)	0.6912 (6)	0.3289 (3)	0.079 (2)	
H18	0.4794	0.7057	0.3601	0.095*	
C19	0.4441 (6)	0.6112 (6)	0.3136 (4)	0.092 (3)	
H19	0.4704	0.5709	0.3343	0.111*	
C20	0.3994 (5)	0.5909 (5)	0.2674 (3)	0.079 (2)	
H20	0.3955	0.5372	0.2560	0.095*	
C21	0.3607 (4)	0.6534 (4)	0.2384 (2)	0.0487 (15)	
C22	0.3089 (4)	0.6366 (4)	0.1895 (2)	0.0505 (15)	
C23	0.2897 (5)	0.5646 (5)	0.1635 (3)	0.070 (2)	
H23	0.3090	0.5128	0.1727	0.083*	
C24	0.2363 (5)	0.5851 (5)	0.1211 (3)	0.073 (2)	
H24	0.2118	0.5498	0.0959	0.088*	
Mn1	0.29740 (5)	0.82270 (6)	0.19825 (4)	0.0462 (2)	
Mo1	0.24323 (3)	0.14506 (3)	0.112281 (17)	0.03994 (14)	
Mo2	0.18961 (3)	0.45615 (3)	−0.01922 (2)	0.04104 (14)	
Mo3	0.42154 (2)	0.31322 (3)	−0.019107 (18)	0.03646 (13)	
Mo4	0.35196 (3)	0.41058 (3)	0.074082 (17)	0.03616 (13)	
Mo5	0.41597 (3)	0.20430 (3)	0.092200 (17)	0.03748 (14)	
Mo6	0.17852 (3)	0.34986 (3)	0.091187 (17)	0.03910 (14)	
N1	0.2007 (3)	0.8748 (3)	0.14048 (17)	0.0390 (11)	
N2	0.2040 (3)	0.8284 (3)	0.23478 (17)	0.0415 (11)	
N3	0.1895 (3)	0.8080 (3)	0.27848 (18)	0.0526 (14)	
H3A	0.2215	0.7891	0.3041	0.063*	
N4	0.3478 (3)	0.9425 (3)	0.2304 (2)	0.0534 (13)	
N5	0.3778 (3)	0.8576 (4)	0.1555 (2)	0.0570 (14)	
N6	0.4054 (4)	0.8223 (4)	0.1200 (3)	0.0742 (18)	
H6	0.3912	0.7755	0.1067	0.089*	
N7	0.3668 (3)	0.7316 (3)	0.2531 (2)	0.0497 (13)	
N8	0.2709 (3)	0.6996 (3)	0.1642 (2)	0.0524 (13)	
N9	0.2268 (3)	0.6667 (4)	0.1234 (2)	0.0609 (15)	
H9A	0.1958	0.6942	0.1011	0.073*	
O1	0.4056 (3)	0.3944 (3)	0.02306 (16)	0.0595 (12)	
O2	0.1489 (2)	0.3974 (3)	0.13557 (15)	0.0558 (12)	
O3	0.2416 (3)	0.0967 (3)	0.16414 (14)	0.0567 (12)	
O4	0.3463 (3)	0.1779 (3)	0.12631 (19)	0.0705 (15)	
O5	0.4037 (3)	0.3167 (3)	0.10546 (17)	0.0636 (13)	
O6	0.2797 (2)	0.3910 (3)	0.10699 (17)	0.0666 (14)	
O7	0.4517 (3)	0.2416 (3)	0.03601 (16)	0.0601 (12)	
O8	0.4019 (3)	0.4847 (3)	0.10750 (16)	0.0588 (12)	
O9	0.4004 (3)	0.2128 (3)	−0.0597 (2)	0.086 (2)	
O10	0.5010 (2)	0.3399 (3)	−0.02956 (19)	0.0588 (12)	
O11	0.2847 (2)	0.4701 (4)	0.02450 (18)	0.0755 (16)	
O12	0.1037 (3)	0.3971 (3)	−0.0559 (2)	0.0782 (17)	
O13	0.1456 (3)	0.1368 (3)	0.0741 (2)	0.086 (2)	
O14	0.1576 (2)	0.4261 (4)	0.03989 (18)	0.0736 (16)	
O15	0.1624 (3)	0.5528 (3)	−0.0271 (2)	0.0646 (13)	
O16	0.2695 (3)	0.0609 (4)	0.0729 (2)	0.0851 (19)	
O17	0.2240 (3)	0.2553 (3)	0.1280 (2)	0.0731 (15)	
O18	0.4931 (2)	0.1832 (3)	0.13411 (16)	0.0629 (13)	
O19A	0.2040 (4)	0.2382 (4)	0.0391 (2)	0.0276 (14)	0.50
O21A	0.3249 (4)	0.2805 (4)	0.0255 (2)	0.0273 (14)	0.50
O19B	0.2906 (4)	0.1881 (4)	0.0389 (2)	0.0281 (14)	0.50
O21B	0.2470 (4)	0.3319 (4)	0.0253 (2)	0.0287 (15)	0.50
O1W	0.1174 (4)	0.7183 (5)	0.0408 (3)	0.107 (2)	
O2W	0.3674 (7)	0.6650 (7)	0.0673 (5)	0.193 (6)	
O3W	0.5347 (7)	0.0708 (9)	0.0266 (7)	0.232 (7)	
P1	0.2500	0.2500	0.0000	0.0222 (3)	

### Atomic displacement parameters (Å^2^)

**Table:**

	*U*^11^	*U*^22^	*U*^33^	*U*^12^	*U*^13^	*U*^23^
C1	0.049 (4)	0.047 (4)	0.047 (3)	−0.001 (3)	0.017 (3)	0.013 (3)
C2	0.062 (4)	0.049 (4)	0.040 (3)	−0.014 (3)	0.008 (3)	0.011 (3)
C3	0.040 (4)	0.058 (4)	0.046 (3)	−0.004 (3)	−0.010 (3)	0.009 (3)
C4	0.038 (3)	0.061 (4)	0.044 (3)	−0.002 (3)	0.007 (3)	0.003 (3)
C5	0.033 (3)	0.038 (3)	0.035 (3)	−0.002 (2)	0.002 (2)	0.000 (2)
C6	0.035 (3)	0.046 (3)	0.039 (3)	0.000 (3)	0.009 (3)	0.003 (3)
C7	0.043 (4)	0.101 (6)	0.051 (4)	0.013 (4)	0.019 (3)	0.018 (4)
C8	0.067 (5)	0.096 (6)	0.041 (3)	−0.001 (4)	0.022 (3)	0.014 (4)
C9	0.063 (4)	0.054 (4)	0.059 (4)	0.003 (4)	0.025 (4)	0.014 (3)
C10	0.080 (6)	0.063 (5)	0.055 (4)	0.001 (4)	0.001 (4)	0.005 (4)
C11	0.092 (6)	0.096 (6)	0.095 (6)	−0.001 (5)	0.001 (5)	0.004 (6)
C12	0.063 (5)	0.075 (6)	0.088 (6)	−0.017 (5)	−0.016 (5)	0.024 (5)
C13	0.031 (3)	0.058 (4)	0.064 (4)	−0.005 (3)	−0.012 (3)	0.023 (3)
C14	0.033 (3)	0.070 (5)	0.059 (4)	−0.001 (3)	0.004 (3)	0.025 (4)
C15	0.055 (4)	0.115 (6)	0.095 (5)	−0.009 (5)	0.006 (4)	0.040 (5)
C16	0.048 (4)	0.159 (10)	0.063 (5)	0.046 (5)	0.026 (4)	0.033 (6)
C17	0.068 (5)	0.059 (5)	0.062 (4)	0.004 (4)	0.000 (4)	0.001 (4)
C18	0.075 (6)	0.079 (6)	0.071 (5)	0.001 (5)	−0.004 (4)	0.011 (5)
C19	0.097 (6)	0.075 (5)	0.086 (5)	0.013 (5)	−0.011 (5)	0.027 (5)
C20	0.085 (6)	0.050 (4)	0.090 (6)	0.014 (4)	0.000 (5)	0.007 (4)
C21	0.046 (4)	0.046 (4)	0.055 (4)	0.009 (3)	0.015 (3)	0.008 (3)
C22	0.050 (4)	0.046 (4)	0.059 (4)	0.007 (3)	0.021 (3)	0.002 (3)
C23	0.094 (6)	0.051 (4)	0.068 (5)	0.015 (4)	0.028 (5)	−0.003 (4)
C24	0.097 (6)	0.060 (5)	0.063 (5)	0.003 (5)	0.022 (5)	−0.018 (4)
Mn1	0.0361 (5)	0.0403 (5)	0.0618 (6)	0.0050 (4)	0.0118 (4)	0.0090 (4)
Mo1	0.0514 (3)	0.0399 (3)	0.0269 (2)	−0.0058 (2)	0.0069 (2)	0.0034 (2)
Mo2	0.0427 (3)	0.0312 (3)	0.0513 (3)	0.0064 (2)	0.0160 (2)	0.0036 (2)
Mo3	0.0249 (3)	0.0423 (3)	0.0419 (3)	−0.0054 (2)	0.0080 (2)	−0.0026 (2)
Mo4	0.0297 (3)	0.0395 (3)	0.0371 (2)	−0.0073 (2)	0.0047 (2)	−0.0095 (2)
Mo5	0.0265 (3)	0.0484 (3)	0.0336 (2)	0.0016 (2)	0.0004 (2)	0.0013 (2)
Mo6	0.0401 (3)	0.0467 (3)	0.0304 (2)	0.0085 (2)	0.0088 (2)	−0.0092 (2)
N1	0.037 (3)	0.038 (3)	0.042 (2)	0.001 (2)	0.010 (2)	0.008 (2)
N2	0.038 (3)	0.047 (3)	0.037 (2)	−0.001 (2)	0.006 (2)	0.006 (2)
N3	0.054 (3)	0.063 (4)	0.036 (2)	−0.003 (3)	0.003 (2)	0.014 (2)
N4	0.044 (3)	0.046 (3)	0.068 (3)	−0.002 (3)	0.010 (3)	0.010 (3)
N5	0.042 (3)	0.067 (4)	0.067 (3)	0.010 (3)	0.021 (3)	0.013 (3)
N6	0.057 (4)	0.082 (5)	0.082 (4)	0.020 (4)	0.014 (4)	0.006 (4)
N7	0.045 (3)	0.045 (3)	0.058 (3)	0.006 (2)	0.011 (3)	0.003 (3)
N8	0.048 (3)	0.051 (3)	0.056 (3)	0.005 (3)	0.008 (3)	−0.001 (3)
N9	0.062 (4)	0.062 (4)	0.053 (3)	0.005 (3)	0.004 (3)	−0.003 (3)
O1	0.092 (3)	0.048 (2)	0.050 (2)	0.022 (2)	0.038 (2)	0.010 (2)
O2	0.048 (3)	0.076 (3)	0.047 (2)	0.002 (2)	0.017 (2)	−0.024 (2)
O3	0.060 (3)	0.073 (3)	0.034 (2)	−0.014 (2)	0.006 (2)	0.016 (2)
O4	0.064 (3)	0.079 (3)	0.083 (3)	−0.037 (3)	0.046 (3)	−0.041 (3)
O5	0.097 (4)	0.052 (3)	0.056 (3)	0.024 (3)	0.045 (3)	0.012 (2)
O6	0.036 (2)	0.105 (4)	0.059 (3)	0.001 (2)	0.012 (2)	0.033 (3)
O7	0.093 (3)	0.050 (3)	0.049 (2)	0.022 (2)	0.039 (2)	0.008 (2)
O8	0.075 (3)	0.049 (3)	0.051 (2)	−0.021 (2)	0.013 (2)	−0.016 (2)
O9	0.087 (4)	0.040 (3)	0.089 (4)	0.011 (3)	−0.053 (3)	−0.015 (3)
O10	0.044 (3)	0.061 (3)	0.080 (3)	−0.005 (2)	0.033 (2)	0.002 (3)
O11	0.041 (3)	0.124 (4)	0.063 (3)	0.008 (3)	0.016 (2)	0.045 (3)
O12	0.076 (3)	0.083 (4)	0.093 (4)	−0.044 (3)	0.054 (3)	−0.048 (3)
O13	0.082 (4)	0.042 (3)	0.092 (4)	0.014 (3)	−0.053 (3)	−0.020 (3)
O14	0.031 (2)	0.126 (4)	0.061 (3)	0.000 (3)	0.007 (2)	0.041 (3)
O15	0.053 (3)	0.042 (3)	0.094 (4)	0.010 (2)	0.010 (3)	0.012 (2)
O16	0.073 (3)	0.096 (4)	0.107 (4)	−0.053 (3)	0.059 (3)	−0.066 (4)
O17	0.065 (3)	0.043 (3)	0.079 (3)	0.003 (2)	−0.038 (3)	−0.013 (2)
O18	0.044 (3)	0.089 (4)	0.043 (2)	0.023 (3)	−0.010 (2)	−0.001 (2)
O19A	0.029 (4)	0.030 (4)	0.023 (3)	−0.002 (3)	0.005 (3)	−0.004 (3)
O21A	0.024 (3)	0.029 (4)	0.029 (3)	−0.002 (3)	0.007 (3)	−0.001 (3)
O19B	0.029 (4)	0.030 (4)	0.023 (3)	0.002 (3)	0.002 (3)	0.001 (3)
O21B	0.027 (4)	0.028 (4)	0.031 (3)	−0.001 (3)	0.007 (3)	−0.002 (3)
O1W	0.086 (5)	0.107 (5)	0.108 (5)	0.011 (4)	−0.012 (4)	−0.006 (4)
O2W	0.207 (11)	0.137 (8)	0.301 (15)	−0.038 (8)	0.187 (12)	−0.053 (9)
O3W	0.169 (11)	0.183 (13)	0.41 (2)	0.001 (9)	0.190 (14)	−0.068 (13)
P1	0.0213 (9)	0.0235 (9)	0.0209 (7)	−0.0005 (7)	0.0036 (7)	−0.0016 (7)

### Geometric parameters (Å, °)

**Table:**

C1—N1	1.342 (7)	Mo1—O19B	2.517 (6)
C1—C2	1.358 (9)	Mo2—O15	1.660 (4)
C1—H1	0.9300	Mo2—O16^i^	1.860 (5)
C2—C3	1.356 (9)	Mo2—O11	1.903 (5)
C2—H2	0.9300	Mo2—O12	1.936 (5)
C3—C4	1.366 (9)	Mo2—O14	1.944 (4)
C3—H3	0.9300	Mo2—O19B^i^	2.471 (7)
C4—C5	1.376 (8)	Mo2—O21B	2.478 (7)
C4—H4	0.9300	Mo3—O10	1.658 (4)
C5—N1	1.344 (7)	Mo3—O1	1.841 (4)
C5—C6	1.463 (7)	Mo3—O7	1.889 (4)
C6—N2	1.324 (7)	Mo3—O13^i^	1.895 (5)
C6—C7	1.394 (8)	Mo3—O9	1.971 (5)
C7—C8	1.375 (9)	Mo3—O19A^i^	2.443 (7)
C7—H7	0.9300	Mo3—O21A	2.510 (6)
C8—N3	1.315 (9)	Mo4—O8	1.662 (4)
C8—H8	0.9300	Mo4—O6	1.852 (4)
C9—N4	1.341 (9)	Mo4—O11	1.882 (5)
C9—C10	1.341 (10)	Mo4—O5	1.906 (5)
C9—H9	0.9300	Mo4—O1	1.954 (4)
C10—C11	1.278 (13)	Mo4—O21B	2.455 (7)
C10—H10	0.9300	Mo4—O21A	2.498 (7)
C11—C12	1.281 (13)	Mo5—O18	1.647 (4)
C11—H11	0.9300	Mo5—O4	1.857 (4)
C12—C13	1.384 (11)	Mo5—O5	1.900 (5)
C12—H12	0.9300	Mo5—O12^i^	1.924 (5)
C13—N4	1.330 (8)	Mo5—O7	1.943 (4)
C13—C14	1.445 (10)	Mo5—O19B	2.461 (7)
C14—N5	1.339 (9)	Mo5—O21A	2.506 (7)
C14—C15	1.428 (11)	Mo6—O2	1.666 (4)
C15—C16	1.490 (14)	Mo6—O9^i^	1.834 (5)
C15—H15	0.9300	Mo6—O14	1.852 (5)
C16—N6	1.360 (11)	Mo6—O17	1.928 (5)
C16—H16	0.9300	Mo6—O6	1.967 (5)
C17—N7	1.325 (9)	Mo6—O19A	2.449 (6)
C17—C18	1.361 (11)	Mo6—O21B	2.506 (6)
C17—H17	0.9300	N2—N3	1.346 (7)
C18—C19	1.370 (12)	N3—H3A	0.8600
C18—H18	0.9300	N5—N6	1.354 (8)
C19—C20	1.377 (12)	N6—H6	0.8600
C19—H19	0.9300	N8—N9	1.331 (8)
C20—C21	1.386 (10)	N9—H9A	0.8600
C20—H20	0.9300	O9—Mo6^i^	1.834 (5)
C21—N7	1.337 (8)	O12—Mo5^i^	1.924 (5)
C21—C22	1.477 (10)	O13—Mo3^i^	1.895 (5)
C22—N8	1.345 (8)	O16—Mo2^i^	1.860 (5)
C22—C23	1.380 (10)	O19A—P1	1.562 (6)
C23—C24	1.376 (11)	O19A—O21A^i^	1.752 (9)
C23—H23	0.9300	O19A—Mo3^i^	2.443 (7)
C24—N9	1.352 (9)	O21A—P1	1.495 (7)
C24—H24	0.9300	O21A—O21B	1.693 (9)
Mn1—N8	2.224 (6)	O21A—O19B	1.723 (9)
Mn1—N5	2.224 (5)	O21A—O19A^i^	1.752 (9)
Mn1—N2	2.250 (5)	O19B—P1	1.531 (6)
Mn1—N4	2.259 (6)	O19B—O21B^i^	1.764 (9)
Mn1—N1	2.260 (5)	O19B—Mo2^i^	2.471 (7)
Mn1—N7	2.283 (5)	O21B—P1	1.519 (7)
Mo1—O3	1.644 (4)	O21B—O19B^i^	1.764 (9)
Mo1—O13	1.880 (5)	P1—O21A^i^	1.495 (7)
Mo1—O16	1.899 (5)	P1—O21B^i^	1.519 (7)
Mo1—O17	1.911 (5)	P1—O19B^i^	1.531 (6)
Mo1—O4	1.960 (5)	P1—O19A^i^	1.562 (6)
Mo1—O19A	2.488 (6)		
			
N1—C1—C2	123.0 (6)	O18—Mo5—O4	102.1 (3)
N1—C1—H1	118.5	O18—Mo5—O5	101.3 (2)
C2—C1—H1	118.5	O4—Mo5—O5	89.4 (2)
C3—C2—C1	119.2 (6)	O18—Mo5—O12^i^	101.7 (3)
C3—C2—H2	120.4	O4—Mo5—O12^i^	89.8 (2)
C1—C2—H2	120.4	O5—Mo5—O12^i^	156.6 (3)
C2—C3—C4	119.1 (6)	O18—Mo5—O7	101.6 (2)
C2—C3—H3	120.5	O4—Mo5—O7	156.3 (2)
C4—C3—H3	120.5	O5—Mo5—O7	86.25 (18)
C3—C4—C5	119.5 (6)	O12^i^—Mo5—O7	85.1 (2)
C3—C4—H4	120.2	O18—Mo5—O19B	159.9 (2)
C5—C4—H4	120.2	O4—Mo5—O19B	65.1 (2)
N1—C5—C4	121.6 (5)	O5—Mo5—O19B	94.2 (2)
N1—C5—C6	115.5 (5)	O12^i^—Mo5—O19B	64.4 (2)
C4—C5—C6	122.9 (5)	O7—Mo5—O19B	92.0 (2)
N2—C6—C7	110.6 (5)	O18—Mo5—O21A	159.5 (2)
N2—C6—C5	119.7 (5)	O4—Mo5—O21A	92.6 (2)
C7—C6—C5	129.8 (6)	O5—Mo5—O21A	64.3 (2)
C8—C7—C6	104.1 (6)	O12^i^—Mo5—O21A	92.4 (3)
C8—C7—H7	127.9	O7—Mo5—O21A	64.6 (2)
C6—C7—H7	127.9	O19B—Mo5—O21A	40.6 (2)
N3—C8—C7	108.2 (6)	O2—Mo6—O9^i^	102.9 (3)
N3—C8—H8	125.9	O2—Mo6—O14	101.7 (3)
C7—C8—H8	125.9	O9^i^—Mo6—O14	91.5 (2)
N4—C9—C10	121.5 (7)	O2—Mo6—O17	100.2 (2)
N4—C9—H9	119.2	O9^i^—Mo6—O17	90.0 (2)
C10—C9—H9	119.2	O14—Mo6—O17	157.2 (2)
C11—C10—C9	121.8 (9)	O2—Mo6—O6	99.7 (2)
C11—C10—H10	119.1	O9^i^—Mo6—O6	157.2 (3)
C9—C10—H10	119.1	O14—Mo6—O6	86.7 (2)
C10—C11—C12	120.1 (11)	O17—Mo6—O6	83.2 (2)
C10—C11—H11	120.0	O2—Mo6—O19A	159.3 (2)
C12—C11—H11	120.0	O9^i^—Mo6—O19A	64.1 (3)
C11—C12—C13	119.9 (10)	O14—Mo6—O19A	95.1 (2)
C11—C12—H12	120.1	O17—Mo6—O19A	65.3 (2)
C13—C12—H12	120.1	O6—Mo6—O19A	93.4 (2)
N4—C13—C12	121.4 (8)	O2—Mo6—O21B	157.4 (2)
N4—C13—C14	117.0 (6)	O9^i^—Mo6—O21B	95.7 (3)
C12—C13—C14	121.6 (7)	O14—Mo6—O21B	64.7 (2)
N5—C14—C15	110.8 (8)	O17—Mo6—O21B	92.5 (2)
N5—C14—C13	117.4 (6)	O6—Mo6—O21B	63.1 (2)
C15—C14—C13	131.8 (8)	O19A—Mo6—O21B	43.1 (2)
C14—C15—C16	103.7 (8)	C1—N1—C5	117.5 (5)
C14—C15—H15	128.2	C1—N1—Mn1	126.3 (4)
C16—C15—H15	128.2	C5—N1—Mn1	116.2 (3)
N6—C16—C15	104.8 (7)	C6—N2—N3	105.7 (5)
N6—C16—H16	127.6	C6—N2—Mn1	115.0 (4)
C15—C16—H16	127.6	N3—N2—Mn1	139.1 (4)
N7—C17—C18	123.2 (7)	C8—N3—N2	111.3 (5)
N7—C17—H17	118.4	C8—N3—H3A	124.3
C18—C17—H17	118.4	N2—N3—H3A	124.3
C17—C18—C19	118.8 (8)	C13—N4—C9	115.3 (6)
C17—C18—H18	120.6	C13—N4—Mn1	115.3 (5)
C19—C18—H18	120.6	C9—N4—Mn1	128.4 (4)
C18—C19—C20	119.7 (8)	C14—N5—N6	107.7 (6)
C18—C19—H19	120.1	C14—N5—Mn1	116.3 (5)
C20—C19—H19	120.1	N6—N5—Mn1	135.9 (5)
C19—C20—C21	117.6 (8)	N5—N6—C16	113.1 (7)
C19—C20—H20	121.2	N5—N6—H6	123.5
C21—C20—H20	121.2	C16—N6—H6	123.5
N7—C21—C20	122.6 (7)	C17—N7—C21	118.1 (6)
N7—C21—C22	116.1 (6)	C17—N7—Mn1	126.2 (5)
C20—C21—C22	121.2 (6)	C21—N7—Mn1	115.7 (4)
N8—C22—C23	110.6 (6)	N9—N8—C22	105.3 (5)
N8—C22—C21	118.2 (6)	N9—N8—Mn1	138.4 (4)
C23—C22—C21	131.3 (6)	C22—N8—Mn1	116.3 (4)
C24—C23—C22	105.8 (7)	N8—N9—C24	112.2 (6)
C24—C23—H23	127.1	N8—N9—H9A	123.9
C22—C23—H23	127.1	C24—N9—H9A	123.9
N9—C24—C23	106.1 (7)	Mo3—O1—Mo4	138.9 (3)
N9—C24—H24	126.9	Mo5—O4—Mo1	139.6 (3)
C23—C24—H24	126.9	Mo5—O5—Mo4	140.0 (3)
N8—Mn1—N5	96.8 (2)	Mo4—O6—Mo6	138.4 (3)
N8—Mn1—N2	96.26 (19)	Mo3—O7—Mo5	138.0 (3)
N5—Mn1—N2	161.7 (2)	Mo6^i^—O9—Mo3	139.2 (4)
N8—Mn1—N4	168.0 (2)	Mo4—O11—Mo2	138.7 (3)
N5—Mn1—N4	73.1 (2)	Mo5^i^—O12—Mo2	136.6 (3)
N2—Mn1—N4	95.04 (19)	Mo1—O13—Mo3^i^	140.9 (4)
N8—Mn1—N1	89.41 (19)	Mo6—O14—Mo2	139.6 (3)
N5—Mn1—N1	93.85 (18)	Mo2^i^—O16—Mo1	142.1 (3)
N2—Mn1—N1	73.49 (17)	Mo1—O17—Mo6	136.3 (3)
N4—Mn1—N1	97.64 (19)	P1—O19A—O21A^i^	53.2 (3)
N8—Mn1—N7	73.6 (2)	P1—O19A—Mo3^i^	124.6 (3)
N5—Mn1—N7	99.5 (2)	O21A^i^—O19A—Mo3^i^	71.3 (3)
N2—Mn1—N7	96.45 (18)	P1—O19A—Mo6	122.9 (4)
N4—Mn1—N7	101.2 (2)	O21A^i^—O19A—Mo6	132.2 (4)
N1—Mn1—N7	159.42 (19)	Mo3^i^—O19A—Mo6	93.6 (2)
O3—Mo1—O13	102.6 (3)	P1—O19A—Mo1	122.3 (4)
O3—Mo1—O16	102.5 (3)	O21A^i^—O19A—Mo1	132.0 (4)
O13—Mo1—O16	89.5 (2)	Mo3^i^—O19A—Mo1	92.4 (2)
O3—Mo1—O17	102.1 (2)	Mo6—O19A—Mo1	92.4 (2)
O13—Mo1—O17	88.8 (2)	P1—O21A—O21B	56.5 (3)
O16—Mo1—O17	155.1 (3)	P1—O21A—O19B	56.3 (3)
O3—Mo1—O4	101.5 (2)	O21B—O21A—O19B	93.5 (4)
O13—Mo1—O4	155.9 (3)	P1—O21A—O19A^i^	56.9 (3)
O16—Mo1—O4	85.4 (2)	O21B—O21A—O19A^i^	92.4 (4)
O17—Mo1—O4	86.2 (2)	O19B—O21A—O19A^i^	91.7 (4)
O3—Mo1—O19A	159.2 (2)	P1—O21A—Mo4	125.2 (4)
O13—Mo1—O19A	62.9 (2)	O21B—O21A—Mo4	68.7 (3)
O16—Mo1—O19A	92.6 (3)	O19B—O21A—Mo4	132.0 (4)
O17—Mo1—O19A	64.6 (2)	O19A^i^—O21A—Mo4	131.5 (4)
O4—Mo1—O19A	93.8 (2)	P1—O21A—Mo5	124.6 (4)
O3—Mo1—O19B	157.9 (2)	O21B—O21A—Mo5	132.9 (4)
O13—Mo1—O19B	94.0 (3)	O19B—O21A—Mo5	68.3 (3)
O16—Mo1—O19B	62.7 (2)	O19A^i^—O21A—Mo5	129.5 (4)
O17—Mo1—O19B	92.6 (2)	Mo4—O21A—Mo5	91.2 (2)
O4—Mo1—O19B	62.7 (2)	P1—O21A—Mo3	124.1 (3)
O19A—Mo1—O19B	42.9 (2)	O21B—O21A—Mo3	129.4 (4)
O15—Mo2—O16^i^	102.5 (3)	O19B—O21A—Mo3	130.9 (4)
O15—Mo2—O11	100.3 (3)	O19A^i^—O21A—Mo3	67.3 (3)
O16^i^—Mo2—O11	90.3 (2)	Mo4—O21A—Mo3	90.4 (2)
O15—Mo2—O12	102.5 (3)	Mo5—O21A—Mo3	91.0 (2)
O16^i^—Mo2—O12	88.4 (2)	P1—O19B—O21A	54.3 (3)
O11—Mo2—O12	156.8 (3)	P1—O19B—O21B^i^	54.3 (3)
O15—Mo2—O14	101.4 (3)	O21A—O19B—O21B^i^	91.3 (4)
O16^i^—Mo2—O14	156.0 (3)	P1—O19B—Mo5	125.4 (4)
O11—Mo2—O14	86.80 (19)	O21A—O19B—Mo5	71.1 (3)
O12—Mo2—O14	85.0 (2)	O21B^i^—O19B—Mo5	134.3 (4)
O15—Mo2—O19B^i^	160.1 (2)	P1—O19B—Mo2^i^	123.7 (3)
O16^i^—Mo2—O19B^i^	64.2 (2)	O21A—O19B—Mo2^i^	134.8 (4)
O11—Mo2—O19B^i^	94.7 (2)	O21B^i^—O19B—Mo2^i^	69.3 (3)
O12—Mo2—O19B^i^	64.1 (2)	Mo5—O19B—Mo2^i^	93.3 (2)
O14—Mo2—O19B^i^	92.3 (2)	P1—O19B—Mo1	122.2 (3)
O15—Mo2—O21B	158.2 (2)	O21A—O19B—Mo1	130.5 (4)
O16^i^—Mo2—O21B	93.1 (3)	O21B^i^—O19B—Mo1	128.6 (4)
O11—Mo2—O21B	64.0 (2)	Mo5—O19B—Mo1	92.0 (2)
O12—Mo2—O21B	93.0 (2)	Mo2^i^—O19B—Mo1	90.9 (2)
O14—Mo2—O21B	64.3 (2)	P1—O21B—O21A	55.1 (3)
O19B^i^—Mo2—O21B	41.8 (2)	P1—O21B—O19B^i^	55.0 (3)
O10—Mo3—O1	102.9 (2)	O21A—O21B—O19B^i^	92.0 (4)
O10—Mo3—O7	101.8 (2)	P1—O21B—Mo4	126.5 (4)
O1—Mo3—O7	90.13 (18)	O21A—O21B—Mo4	71.4 (3)
O10—Mo3—O13^i^	101.3 (3)	O19B^i^—O21B—Mo4	135.1 (4)
O1—Mo3—O13^i^	90.7 (2)	P1—O21B—Mo2	123.9 (4)
O7—Mo3—O13^i^	156.1 (3)	O21A—O21B—Mo2	132.7 (4)
O10—Mo3—O9	100.4 (3)	O19B^i^—O21B—Mo2	68.9 (3)
O1—Mo3—O9	156.7 (3)	Mo4—O21B—Mo2	91.7 (2)
O7—Mo3—O9	85.2 (2)	P1—O21B—Mo6	121.7 (3)
O13^i^—Mo3—O9	84.7 (2)	O21A—O21B—Mo6	131.9 (4)
O10—Mo3—O19A^i^	157.1 (2)	O19B^i^—O21B—Mo6	127.0 (4)
O1—Mo3—O19A^i^	94.8 (2)	Mo4—O21B—Mo6	92.0 (2)
O7—Mo3—O19A^i^	92.4 (2)	Mo2—O21B—Mo6	91.3 (2)
O13^i^—Mo3—O19A^i^	63.7 (2)	O21A—P1—O21A^i^	180.0 (8)
O9—Mo3—O19A^i^	62.7 (2)	O21A—P1—O21B	68.4 (4)
O10—Mo3—O21A	161.3 (2)	O21A^i^—P1—O21B	111.6 (4)
O1—Mo3—O21A	65.5 (2)	O21A—P1—O21B^i^	111.6 (4)
O7—Mo3—O21A	65.1 (2)	O21A^i^—P1—O21B^i^	68.4 (4)
O13^i^—Mo3—O21A	93.7 (3)	O21B—P1—O21B^i^	180.0 (5)
O9—Mo3—O21A	92.0 (3)	O21A—P1—O19B^i^	110.6 (4)
O19A^i^—Mo3—O21A	41.4 (2)	O21A^i^—P1—O19B^i^	69.4 (4)
O8—Mo4—O6	103.8 (2)	O21B—P1—O19B^i^	70.6 (3)
O8—Mo4—O11	102.0 (3)	O21B^i^—P1—O19B^i^	109.4 (3)
O6—Mo4—O11	90.04 (19)	O21A—P1—O19B	69.4 (4)
O8—Mo4—O5	100.6 (2)	O21A^i^—P1—O19B	110.6 (4)
O6—Mo4—O5	89.7 (2)	O21B—P1—O19B	109.4 (3)
O11—Mo4—O5	156.8 (3)	O21B^i^—P1—O19B	70.6 (3)
O8—Mo4—O1	100.0 (2)	O19B^i^—P1—O19B	180.0 (8)
O6—Mo4—O1	156.2 (2)	O21A—P1—O19A^i^	69.9 (3)
O11—Mo4—O1	85.7 (2)	O21A^i^—P1—O19A^i^	110.1 (3)
O5—Mo4—O1	85.27 (18)	O21B—P1—O19A^i^	107.6 (3)
O8—Mo4—O21B	161.8 (2)	O21B^i^—P1—O19A^i^	72.4 (3)
O6—Mo4—O21B	65.5 (2)	O19B^i^—P1—O19A^i^	72.6 (3)
O11—Mo4—O21B	64.7 (2)	O19B—P1—O19A^i^	107.4 (3)
O5—Mo4—O21B	94.2 (2)	O21A—P1—O19A	110.1 (3)
O1—Mo4—O21B	91.7 (2)	O21A^i^—P1—O19A	69.9 (3)
O8—Mo4—O21A	158.2 (2)	O21B—P1—O19A	72.4 (3)
O6—Mo4—O21A	92.4 (2)	O21B^i^—P1—O19A	107.6 (3)
O11—Mo4—O21A	92.5 (3)	O19B^i^—P1—O19A	107.4 (3)
O5—Mo4—O21A	64.4 (2)	O19B—P1—O19A	72.6 (3)
O1—Mo4—O21A	64.5 (2)	O19A^i^—P1—O19A	180.0 (4)
O21B—Mo4—O21A	40.0 (2)		

Symmetry codes: (i) −*x*+1/2, −*y*+1/2, −*z*.

### Hydrogen-bond geometry (Å, °)

**Table:**

*D*—H···*A*	*D*—H	H···*A*	*D*···*A*	*D*—H···*A*
N9—H9A···O1W	0.86	1.96	2.786 (9)	160
N6—H6···O2W	0.86	2.10	2.951 (13)	171
N3—H3A···O17^ii^	0.86	1.97	2.814 (7)	165

Symmetry codes: (ii) −*x*+1/2, *y*+1/2, −*z*+1/2.

## References

[bb1] Artero, V. & Proust, A. (2000). *Eur. J. Inorg. Chem.* pp. 2393–2400

[bb2] Bruker (2001). *SAINT-Plus* and *SADABS* Bruker AXS Inc., Madison, Wisconsin, USA.

[bb3] Bruker (2004). *APEX2* Bruker AXS Inc., Madison, Wisconsin, USA.

[bb4] Kurmoo, M., Bonamico, M., Bellitto, C., Fares, V., Federici, F., Guionneau, P., Ducasse, L., Kitagawa, H. & Day, P. (1998). *Adv. Mater.* **7**, 545–550.

[bb5] Niu, J. Y., Shan, B. Z. & You, X. Z. (1999). *Transition Met. Chem.* **24**, 108–114.

[bb6] Pope, M. T. & Müller, A. (1991). *Angew. Chem. Int. Ed.* **30**, 34–38.

[bb7] Sheldrick, G. M. (2008). *Acta Cryst.* A**64**, 112–122. 10.1107/S0108767307043930 18156677

[bb8] Yang, W. B., Lu, C. Z., Wu, C. D. & Zhuang, H. H. (2003). *Chin. J. Struct. Chem.* **22**, 137–142.

[bb9] Zhang, X. T., Dou, J. M., Wei, P. H., Li, D. C., Li, B., Shi, C. W. & Hu, B. (2009). *Inorg. Chim. Acta*, **362**, 3325–3332.

[bb10] Zhang, X. T., Wei, P. H., Sun, D. F., Ni, Z. H., Dou, J. M., Li, B., Shi, C. W. & Hu, B. (2009). *Cryst. Growth Des.* **9**, 4424–4428.

